# Alcohol Use Disorder and Alcohol-Associated Liver Disease

**DOI:** 10.35946/arcr.v42.1.13

**Published:** 2022-11-10

**Authors:** Resham Ramkissoon, Vijay H. Shah

**Affiliations:** Department of Gastroenterology and Hepatology, Mayo Clinic, Rochester, Minnesota

**Keywords:** alcohol, alcohol-associated liver disease, screening, prevention, mortality, patient readmission, policy, liver diseases

## Abstract

This article is part of a Festschrift commemorating the 50th anniversary of the National Institute on Alcohol Abuse and Alcoholism (NIAAA). Established in 1970, first as part of the National Institute of Mental Health and later as an independent institute of the National Institutes of Health, NIAAA today is the world’s largest funding agency for alcohol research. In addition to its own intramural research program, NIAAA supports the entire spectrum of innovative basic, translational, and clinical research to advance the diagnosis, prevention, and treatment of alcohol use disorder and alcohol-related problems. To celebrate the anniversary, NIAAA hosted a 2-day symposium, “Alcohol Across the Lifespan: 50 Years of Evidence-Based Diagnosis, Prevention, and Treatment Research,” devoted to key topics within the field of alcohol research. This article is based on Dr. Shah’s presentation at the event. NIAAA Director George F. Koob, Ph.D., serves as editor of the Festschrift.

Alcohol use disorder (AUD) is prevalent worldwide, and the burden of heavy alcohol consumption has been increasing over time. An important complication of prolonged, heavy alcohol use is alcohol-associated liver disease (ALD), which can progress from liver steatosis to fibrosis and cirrhosis and frequently involves alcohol-associated hepatitis. In particular, cirrhosis—the most severe type of ALD—can be associated with fatal and resource-intensive complications and impose a significant social and financial burden on families, hospitals, and communities.

This article summarizes the epidemiology of alcohol use and ALD and describes the outcomes and mortality associated with ALD. This is followed by a review of screening and prevention approaches for AUD and ALD, as well as of current treatment strategies for both conditions, including integrated treatment approaches. Policy measures to mitigate the impact of alcohol misuse are also discussed.

## Epidemiology of Alcohol Use and ALD

There is currently a very high burden of alcohol use and misuse globally. In 2016, an estimated 2.4 billion people worldwide consumed alcohol, including 1.5 billion men and 900 million women.^1^ Furthermore, nearly 40% of people who consume alcohol reported heavy, episodic drinking in 2016 (defined as 60 or more grams of pure alcohol on at least one single occasion at least once per month).^2^

According to the fifth edition of the *Diagnostic and Statistical Manual of Mental Disorders*, AUD is a maladaptive pattern of alcohol use characterized by two or more from a list of symptoms, such as increasing alcohol use despite negative consequences; persistent, unsuccessful attempts to quit drinking; craving; tolerance; or withdrawal.^3^ The estimated global prevalence of AUD is currently 9% and continues to rise.^4^ Of note, psychiatric comorbidities are often present in individuals with AUD and may precede the onset of heavy alcohol use.^5^

**Figure 1:**
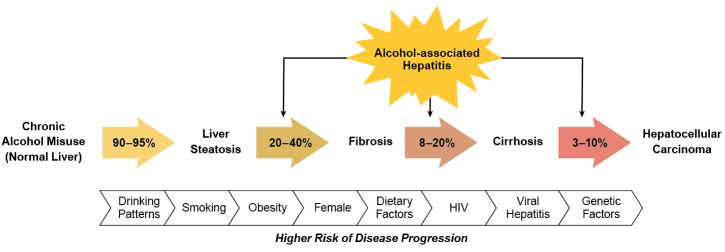
The spectrum of alcohol-associated liver disease, from steatosis to cirrhosis complicated by hepatocellular carcinoma Alcohol-associated hepatitis can occur at any stage of disease. Numerous risk factors and comorbidities contribute to the risk of disease progression.^6^ Note: HIV, human immunodeficiency virus.

ALD is a common complication associated with long-term alcohol misuse and AUD, and clinicians may encounter a spectrum of ALD in practice (Figure 1). Hepatic steatosis occurs in 90% to 95% of patients with chronic, heavy alcohol use. Steatosis causes inflammation of the liver, known as steatohepatitis, and progression to liver fibrosis occurs in 20% to 40% of patients. Liver fibrosis can continue to progress and result in cirrhosis in 8% to 20% of patients. Hepatocellular carcinoma is a primary liver neoplasm that is a complication of cirrhosis, occurring in 3% to 10% of these patients.^6^ Alcohol-associated hepatitis is a specific clinical entity that occurs with long-term heavy alcohol use and may occur anywhere along the spectrum of ALD. There are several risk factors for progression of ALD, which include female sex, obesity, dietary factors, genetic polymorphisms, harmful patterns of alcohol consumption, and smoking. Clinicians should also consider and treat comorbidities that may contribute to disease progression, such as viral hepatitis, hemochromatosis, and human immunodeficiency virus (HIV).^6^

Cirrhosis is associated with chronic alcohol use, which accounts for 21% of physiologically compensated cirrhosis around the world. The global prevalence of alcohol-related, compensated cirrhosis remained relatively unchanged from 1990 (290/100,000 people) to 2017 (288/100,000 people). However, the global prevalence of decompensated cirrhosis rose from 1.1 million individuals in 1990 to 2.5 million individuals in 2017, with the greatest increases found in Western and Central Europe. Furthermore, ALD is the underlying cause of 30% of hepatocellular carcinoma cases.^7^ The overall burden of ALD is expected to increase over time. This prediction is based on multiple variables, including socioeconomic factors, changes in drinking patterns, and the rising prevalence of obesity and fatty liver disease.^5^

## Mortality and Outcomes Associated With ALD

The incidence of cirrhosis is expected to triple by the year 2030 due to the rising prevalence of ALD as well as non-alcoholic fatty liver disease (NAFLD). Cirrhosis is associated with fatal complications, such as gastrointestinal hemorrhage, renal failure, and hepatocellular carcinoma, which impose significant social and financial burdens on families, hospitals, and communities. Mortality rates from cirrhosis have risen in the United States from 2009 to 2016, with the greatest relative increase observed in young people (ages 25 to 34).^8^ This trend parallels increased mortality due to AUD. Compared with women, men had a higher age-adjusted mortality due to cirrhosis (2:1) and hepatocellular carcinoma (4:1). However, women experienced a more rapid increase in cirrhosis-related mortality than did men; the annual percentage increase in mortality was highest in women ages 25 to 34.^8^ Among different racial/ethnic groups in the United States, Native Americans and white Americans had the highest mortality due to cirrhosis, whereas Asians and Pacific Islanders had the highest mortality due to hepatocellular carcinoma. Furthermore, Hispanic individuals had a higher mortality from cirrhosis and hepatocellular carcinoma, compared with non-Hispanic individuals.^8^

The development of ALD may also be dependent on other factors related to the patient’s health, such as obesity. Dietary guidelines by the U.S. government state that to minimize risks associated with drinking, adults of legal drinking age can choose not to drink or to drink in moderation by limiting intake to two drinks or less per day for men and one drink or less per day for women, on days when alcohol is consumed.^9^ However, the American College of Gastroenterology recommends that the obese population should avoid alcohol consumption entirely due to increased risk of hepatic steatosis—a condition characterized by lipid deposits within the liver that is caused by heavy alcohol consumption or metabolic syndrome and can lead to chronic liver disease and cirrhosis.^10^ A large cohort study using the Mayo Clinic Biobank examined the impact of alcohol consumption and obesity on the development of hepatic steatosis and mortality.^11^ Moderate alcohol consumption (defined in the study as no more than two standard drinks per day) increased the risk of hepatic steatosis and all-cause mortality in obese individuals (body mass index [BMI] > 30 kg/m^2^), whereas heavy drinking (defined as more than two standard drinks per day) increased the risk of hepatic steatosis and all-cause mortality in all patients, regardless of BMI.^11^ In individuals with a normal BMI (< 25 kg/m^2^), moderate alcohol consumption lowered the risk of hepatic steatosis and all-cause mortality. This effect was not observed in overweight individuals (BMI 25 kg/m^2^ to 30 kg/m^2^).

## Screening and Prevention Strategies for AUD and ALD

The key to mitigating the future burden of AUD and ALD is early detection and prevention. Unfortunately, ALD is often detected at a later stage of disease when patients present with decompensated cirrhosis. Improved screening modalities for liver fibrosis are needed to identify affected individuals before irreversible, decompensated liver disease develops. Technologies such as smartphone applications, telemedicine, or electronic medical records can be used to improve population screening for AUD and ALD and may prove useful in linking people with a diagnosis of AUD or ALD to treatment programs or support groups. Such tools have been well received by individuals who drink heavily and those who have cirrhosis.^5^

Not all people with AUD are identified through screening or receive treatment. Barriers to AUD treatment include a shortage of providers, limited insurance reimbursement, and patient attitude toward treatment. Most screening for AUD occurs in health care environments, usually when patients are evaluated for other medical issues. Individuals who have little to no contact with health care systems almost never receive screening.^5^

Accordingly, in-person screening for AUD and ALD should be expanded outside of traditional health care environments to nontraditional settings such as pharmacies, annual employee health screenings, or driver’s license renewal appointments.^5^ A prime example for this approach is the effectiveness of screening for hypertension at community barbershops.^12^

## Treatment of AUD

Once AUD or ALD has been identified, treatment and therapy should be initiated early to prevent disease progression or relapse to alcohol use. Treatment of AUD may involve nonpharmacological and pharmacological approaches.

### Nonpharmacological Treatment

Nonpharmacological therapies for AUD, such as patient counseling and motivational interviewing, play a key role in achieving alcohol abstinence. These strategies are used universally and can be employed by any health care provider, including primary care providers.

Motivational interviewing is a form of nonconfrontational counseling that encourages patients to make choices consistent with their long-term goals and health. This technique is especially helpful in patients with heavy alcohol use that does not meet diagnostic criteria for AUD.^13^ Providing patients with feedback surrounding changes in liver tests is associated with decreased alcohol use in patients who have, or are at risk of, chronic liver disease.^14^ Motivational interviewing can be used in combination with pharmacotherapy to help patients achieve alcohol abstinence.^15^ Other nonpharmacological treatment strategies for AUD include establishing a supportive patient-physician relationship, scheduling follow-up clinic visits, engaging family members for support, referring patients to 12-step programs, developing coping strategies to manage early relapse, and treating psychiatric comorbidities.^15^

### Pharmacological Treatment

The U.S. Food and Drug Administration (FDA) has approved three medications to treat AUD; these include disulfiram, naltrexone, and acamprosate. Baclofen is another option for therapy; however, it has not been approved by FDA. Although these medications are well studied for AUD, few studies have examined their effectiveness in patients with cirrhosis. Any medication approved by FDA can be used in patients with mild forms of liver disease; however, the use of disulfiram and naltrexone is cautioned in patients with cirrhosis or any features suggestive of liver dysfunction.^15^

Disulfiram is an acetaldehyde dehydrogenase inhibitor that produces an acetaldehyde syndrome characterized by facial flushing, nausea, vomiting, tachycardia, and hypotension when consumed with alcohol. It is prescribed as a deterrent to alcohol consumption based on this reaction. A meta-analysis showed that disulfiram significantly helped with alcohol abstinence in six out of 11 clinical trials.^16^ Disulfiram is most effective in patients who are committed to abstinence or take it in a monitored fashion.^16^ Cirrhosis is a known contraindication to disulfiram use due to reported events of liver failure leading to death or liver transplantation. Liver toxicity also has been reported in patients without liver disease.

Naltrexone is an opioid receptor antagonist that affects alcohol use primarily by inhibiting mu-opioid receptors and reducing the rewarding and reinforcing effects of alcohol. Clinical trials have demonstrated that naltrexone therapy is associated with a reduced risk of relapse to alcohol use and longer abstinence compared to placebo.^17^ Naltrexone can result in elevated liver enzymes, especially at doses greater than 100 mg per day, and should be avoided in patients with acute hepatitis or acute liver failure. Providers should monitor for injection-site hematomas related to naltrexone injections in patients with coagulopathy of liver disease. Naltrexone is contraindicated in patients who are being treated for opioid use disorder with mu-opioid receptor agonists (i.e., methadone or buprenorphine).

Acamprosate can reduce the symptoms of alcohol craving during prolonged abstinence and reduces alcohol intake in patients with AUD.^18^ Its therapeutic effects on AUD are thought to be through antagonizing *N*-methyl-D-aspartate (NMDA) receptors, although it also has been reported that pharmacological effects could modulate gamma-aminobutyric acid type A (GABA_A_) receptor activity.^15^ Acamprosate can be used safely in patients undergoing treatment for opioid use disorder and has no hepatic metabolism. However, its safety and efficacy in patients with advanced liver disease has not been validated. Dose adjustments of acamprosate are required in patients with chronic kidney disease, especially when the creatinine clearance is below 30 mL per minute.

Baclofen is a selective GABA type B (GABA_B_) receptor antagonist that is typically prescribed for muscle spasticity. Although it is not approved by FDA for treatment of AUD, baclofen is commonly used off-label in other countries. Several clinical trials and open-label studies using baclofen to treat AUD in patients with advanced liver disease have shown mixed results.^19^,^20^ Overall, baclofen use is not associated with liver toxicity and can be used safely in patients with ALD.^15^

## Integrated Care of Patients With AUD and ALD

In patients with alcohol-associated hepatitis, the most important predictor of long-term mortality is alcohol relapse. In fact, recurrent episodes of alcohol-associated hepatitis in patients who relapse to alcohol use have a mortality of nearly 60%. Among patients with alcohol-associated hepatitis, 34% to 37% relapse to alcohol use, and approximately 30% are readmitted to hospitals. The most common reasons for readmission are recurrent alcohol-associated hepatitis (19%) and alcohol intoxication and/or alcohol withdrawal (8%).^21^

Integrated treatment that addresses not only the patients’ liver disease but also alcohol use can improve outcomes. In patients with alcohol-associated hepatitis, alcohol rehabilitation—defined as residential or outpatient AUD treatment or mutual support group participation—after hospital discharge is associated with a 70% to 84% decrease in 30-day readmission rate, an 89% to 91% decrease in 30-day alcohol relapse, and an 80% reduction in mortality.^21^ Furthermore, alcohol rehabilitation plays a particularly important role in therapy of people with AUD and ALD because only a few medications to treat AUD can be used in individuals with recent or active alcohol-associated hepatitis. A large body of evidence suggests that psychosocial interventions, such as cognitive behavioral therapy and motivational interviewing, are effective tools for supporting alcohol abstinence.

**Figure 2:**
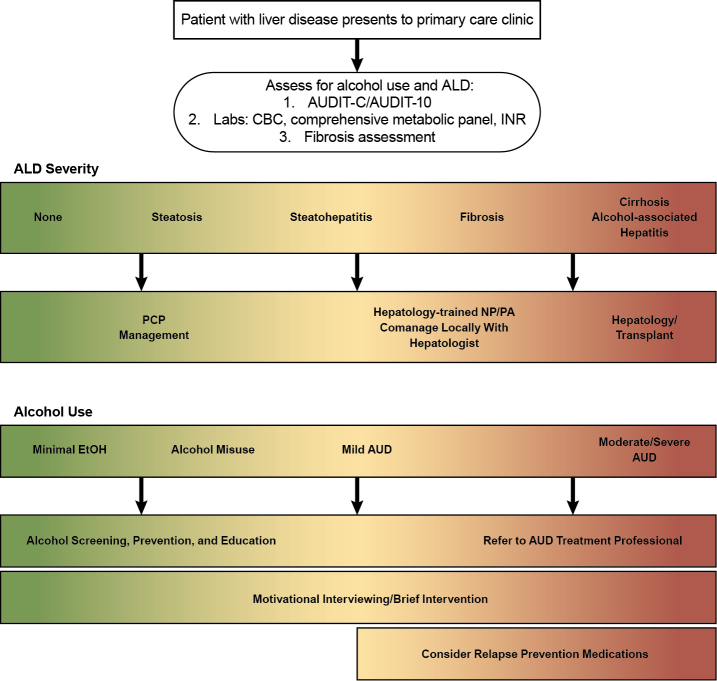
Treatment paradigm for patients with AUD and the spectrum of ALD Reprinted with permission from Asrani et al., 2021.^5^ *Note*: ALD, alcohol-associated liver disease; AUD, alcohol use disorder; AUDIT-10, 10-item Alcohol Use Disorders Identification Test; AUDIT-C, three-item Alcohol Use Disorders Identification Test; CBC, complete blood count; EtOH, ethanol; INR, international normalized ratio; NP, nurse practitioner; PA, physician assistant; PCP, primary care provider.

Overall, there is a clear need for the implementation of alcohol rehabilitation in preventing undesirable patient outcomes.^21^ Currently, only 16% to 20% of patients with alcohol-associated hepatitis attend alcohol rehabilitation. However, patients who were seen by addiction specialists during hospitalization are twice as likely to attend alcohol rehabilitation after discharge.^21^ Implementing these strategies for the care of patients with AUD can reduce the risk of alcohol relapse, recurrent alcohol-associated hepatitis, hospital readmission, and overall mortality. Therefore, it is strongly suggested that health care providers should arrange for alcohol rehabilitation at the index hospitalization, and referral should be used as a quality metric in the management of all patients with alcohol-associated hepatitis. In this manner, implementing quality metrics could lead to improved patient outcomes.^21^ Further integrated care can include evaluation of alcohol biomarkers, validated screening tools, appropriate pharmacotherapy, multidisciplinary and telehealth care, as well as appropriate referral for specialty care (Figure 2).

Recent technological advances have improved health care delivery to patients with AUD and ALD. Biomonitoring (using wearable devices) and telehealth have revolutionized patients’ access to health care. With these approaches, providers can obtain clinical information, such as blood alcohol levels or vital signs, and respond accordingly through smartphone applications and other technology. This advancement has allowed providers to reach more patients.^22^

## Treatment of ALD

The mainstay of treatment for patients with alcohol-associated hepatitis is therapy for AUD, either pharmacologic, nonpharmacologic, or a combination thereof. Alcohol-associated hepatitis is classified as either mild or severe based on the Maddrey discriminant function (mDF) or the model for end-stage liver disease (MELD) scores.^23^ Therapy for patients with mild alcohol-associated hepatitis (mDF < 32 or MELD < 20) is centered around supportive care and AUD therapy. Nutritional support is essential as malnutrition and sarcopenia are common complications of ALD and have a negative impact on patient outcomes. Enteral nutrition supplementation, instead of intravenous administration, is preferred due to lower cost, greater safety, and lower risk of infection. Feeding tube insertion is safe in patients with nonbleeding, esophageal varices who have not undergone recent variceal band ligation. Fluid resuscitation, preferably with albumin, is also part of treatment.^10^

For patients with severe alcohol-associated hepatitis (mDF > 32 and MELD > 20), corticosteroids should be considered in addition to supportive therapy. If there are no contraindications to corticosteroids, prednisolone can be initiated to treat severe alcohol-associated hepatitis as its use modestly increases 1-month survival.^24^ Corticosteroid use in clinical practice is often limited by concern about adverse reactions and high risk of infection. Once treatment has been started, clinicians should assess patient response using the Lille score, which is a calculated score on treatment day 7 to estimate if a patient is responding to corticosteroid therapy.^25^ Clinicians can discontinue corticosteroids in nonresponders and avoid the increased risk of infection associated with their use. There is some indication that the Lille score on day 4 is as accurate as on day 7 in predicting treatment response.^25^ There is currently an unmet need for alternative and safe medical therapy for severe alcohol-associated hepatitis.^10^

### Liver Transplantation

Liver transplantation is a treatment option for patients with severe ALD, including those with severe alcohol-associated hepatitis that fails to respond to corticosteroids. ALD is the leading indication for liver transplant in the United States, accounting for 15% of liver transplants in the nation, as well as for 20% of liver transplants in Europe.^10^,^26^ The process starts with a referral to a liver transplant center, followed by a formal evaluation and listing for transplant. However, numerous barriers to receiving a liver transplant exist for patients with ALD. For example, physicians may be biased against referral for a formal evaluation based on patient age or race, lack of empathy due to considering AUD a behavior rather than a disease, duration of alcohol use, and geographical area.^27^

Relapse to alcohol use occurs in 17% to 30% of patients on a waiting list for a liver transplant and in 10% to 60% of post-transplant patients.^28^ This emphasizes that a liver transplant cures liver disease but not the underlying AUD. Many transplant programs require patients to abstain from alcohol for a minimum of 6 months before considering a liver transplant; however, protracted abstinence is not a reliable predictor of recidivism. Instead, important predictors include age, social support, psychiatric comorbidities, polysubstance abuse, family history, and previous failed rehabilitation attempts. The Psychosocial Assessment of Candidacy for Transplantation scale is widely used to determine a patient’s risk of recidivism and need for alcohol rehabilitation prior to liver transplantation.^29^ Patients should be screened for recidivism at every clinic visit, as 10-year survival after liver transplantation is 45% to 71% in those with harmful alcohol use versus 75% to 93% in abstinent patients with occasional slips.^30^ Self-reported alcohol use may not be reliable, and clinicians should consider using biomarkers to assess for ongoing alcohol consumption.^10^

Liver transplantation for ALD remains a controversial topic and requires careful consideration and expertise. Established criteria for transplant candidacy specify that patients should be presenting with liver disease for the first time, have failed medical therapy, and are without severe medical or psychosocial comorbidities. It is important to avoid liver transplantation in patients who will recover without it and in those with low predicted short-term survival. This will avoid creating a disparity in available liver grafts based on indication and socioeconomic factors. Transplant candidates with ALD should have a high likelihood of long-term abstinence, and treatment of AUD should be incorporated into pre- and post-transplant care.^31^

### Recent Advances in ALD and Implications for Treatment

Alcohol-associated hepatitis is characterized by unrelenting inflammation that is a complex response to hepatocellular stress and death. Advancements in understanding the molecular biology of ALD have changed approaches to caring for patients. Heavy, long-term consumption of alcoholic beverages results in damage to hepatocytes, which respond by releasing extracellular vesicles (EVs). The release of EVs results in activation of inflammatory cells (e.g., macrophages), which release inflammatory cytokines such as tumor necrosis factor alpha (TNF-alpha), interleukin 1 beta (IL-1-beta ), and IL-6.^32^ Research is now being conducted to investigate the interplay of other hepatic endothelial cells, hepatic stellate cells, and the patient’s inflammatory cascade of lymphocytes. Further research also is needed to determine how alcohol’s effects on the intestine may result in mild intestinal injury, alter intestinal permeability, and affect the gut microbiome, which can result in the progression of ALD.^32^

EV release from hepatocytes, which has been observed with in vitro studies, mouse models, and human subjects in response to liver injury, may be useful as a biomarker for ALD. Sehrawat et al. examined the quantity of EVs released and demonstrated that a high EV count was associated with a worse prognosis for ALD compared to a low EV count, and was predictive of disease severity and mortality.^33^ Furthermore, detectable EVs in the blood were liver-specific and could be useful in the diagnosis of ALD and dynamic risk profiling.^33^ Magnetic resonance elastography is also under investigation as a possible diagnostic tool for assessing inflammation, hepatic injury, and fibrosis in ALD.^34^ This technology could be useful in clinical practice and avoid the need for a liver biopsy and its associated risks.

Enhanced understanding of the molecular biology of ALD has revealed targetable disease mechanisms for drug therapies and promising alternatives to corticosteroid therapy. Some therapies under current investigation include granulocyte colony stimulating factor (G-CSF), the IL-1 receptor antagonist anakinra, IL-22, and high-dose vitamin C. IL-22 therapy is of notable interest as it has already succeeded in a proof-of-concept study.^35^ Thus, IL-22 reduced hepatocyte injury, promoted liver regeneration, reduced steatosis and fibrosis, and was not immunosuppressive. Recombinant IL-22 (termed F-652 in clinical trials) has demonstrated safety and efficacy in early, open-label studies with improved MELD scores and Lille scores, as well as reduced inflammatory markers. F-652 administered to patients with moderate to severe alcohol-associated hepatitis was associated with a reduction in patient MELD score at days 28 and 42.^35^ Further studies are being conducted to evaluate the real-world efficacy of F-652. Other cytokines, such as TNF-alpha or transcription factor BRD4, also may be targeted to reduce hepatocellular injury.^35^

## Policies to Mitigate the Impact of AUD and ALD

The effects of AUD and ALD have major individual and societal impacts. National and regional interventions can help decrease the societal impact and reduce the number of individuals at risk. To lower the overall burden associated with AUD and ALD, medical societies have recommended community-wide alcohol reduction strategies as well as personalized treatment options for these conditions. Various initiatives led by the World Health Organization also aim to decrease the impact of alcohol use, for example, through appropriate taxation of alcohol, restricted alcohol availability, and restricted promotion to vulnerable populations.^5^

One of the strongest approaches to influencing alcohol consumption and, consequently, ALD risk at the population level is regulation of the unit price of alcohol through measures such as alcohol taxation. When alcohol prices increase, alcohol consumption and ALD burden notably decrease. Conversely, reduced alcohol prices are associated with increased alcohol consumption and alcohol-related deaths. However, the impact of these measures varies among population subgroups and is most prominent in groups with the highest amount of alcohol use and those with lower socioeconomic status.^36^,^37^ In addition to taxation, strategies such as adjusting for inflation and income, minimal pricing policies, volumetric taxes, and banning volume discounts can be employed to reduce alcohol consumption.^5^

Reducing availability is another strategy to decrease alcohol consumption and its consequences at the population level. Regulating hours of alcohol sales, controlling liquor licenses, and raising minimum legal purchasing age are examples of strategies to reduce alcohol availability. Educational initiatives also have proved effective in reducing the per-capita alcohol consumption. For example, over a period of 20 years, Iceland was able to reduce alcohol and drug use in young people from 42% to 5% by introducing a wide range of targeted policies involving families, schools, communities, and politicians.^38^ Finally, limiting alcohol-related marketing, particularly to vulnerable populations such as youth, is an important strategy to reduce alcohol consumption.^5^

## Conclusions

AUD and ALD are prevalent worldwide and are associated with significant morbidity and mortality. Currently, the individuals at highest risk of mortality are young people, women, as well as Native Americans and white Americans. Expanded screening approaches can reach individuals at high risk and those who have little contact with health care systems.

Treatment of the underlying AUD is essential for improving outcomes of patients with ALD. There are several approved medications for AUD; however, their use is cautioned in people with advanced liver disease. Alcohol rehabilitation significantly reduces 30-day hospital readmission, alcohol relapse, and mortality in individuals with ALD. Consulting addiction specialists and setting up alcohol rehabilitation at hospital discharge are quality metrics used when managing hospitalized patients with ALD.

Treatment of alcohol-associated hepatitis is centered around therapy for AUD, as well as supportive medical therapies and nutrition. Corticosteroids improve 1-month survival in alcohol-associated hepatitis, but the potential side effects limit their use. Additionally, liver transplantation is an option for patients with severe alcohol-associated hepatitis and advanced liver disease who have failed other therapies. Listing a patient for transplant requires a formal evaluation at a liver transplant center; moreover, health care providers should screen for ongoing alcohol use at every clinic visit, both while patients are wait-listed and after liver transplantation. In recent years, several advancements in ALD research have led to improved diagnosis, prognostication, and treatment. For example, recombinant human IL-22 is an emerging therapy that is being tested in clinical trials for the treatment of alcohol-associated hepatitis. Additionally, policy makers have an opportunity to expand regulations to help reduce the burden of heavy alcohol consumption and, consequently, ALD.
